# YOLOX-based blue laser weeding robot in corn field

**DOI:** 10.3389/fpls.2022.1017803

**Published:** 2022-11-04

**Authors:** Huibin Zhu, Yuanyuan Zhang, Danlei Mu, Lizhen Bai, Hao Zhuang, Hui Li

**Affiliations:** ^1^ College of Modern Agricultural Engineering, Kunming University of Science and Technology, Kunming, China; ^2^ Shandong Academy of Agricultural Machinery Science, Jinan, China

**Keywords:** deep learning, laser weeding, weed recognition, weeding robot, Yolo algorithm

## Abstract

A YOLOX convolutional neural network-based weeding robot was designed for weed removal in corn seedling fields, while verifying the feasibility of a blue light laser as a non-contact weeding tool. The robot includes a tracked mobile platform module, a weed identification module, and a robotic arm laser emitter module. Five-degree-of-freedom robotic arm designed according to the actual weeding operation requirements to achieve precise alignment of the laser. When the robot is in operation, it uses the texture and shape of the plants to differentiate between weeds and corn seedlings. The robot then uses monocular ranging to calculate the coordinates of the weeds using the triangle similarity principle, and it controls the end actuator of the robotic arm to emit the laser to kill the weeds. At a driving speed of 0.2 m·s^-1^ on flat ground, the weed robot’s average detection rate for corn seedlings and weeds was 92.45% and 88.94%, respectively. The average weed dry weight prevention efficacy was 85%, and the average seedling injury rate was 4.68%. The results show that the robot can accurately detect weeds in corn fields, and the robotic arm can precisely align the weed position and the blue light laser is effective in removing weeds.

## 1 Introduction

Weeds are an important factor affecting maize yield, limiting yield and quality by competing for nutrients, sunlight, and space (Rajcan and Swanton, 2001; Ab Rahman et al., 2018; Westwood et al., 2018), and the main methods of weed control in maize fields are currently biological control, chemical weed control, mechanical weed control, and physical weed control. The advantage of biological control is that it is less disruptive to agroecosystems (Barratt et al., 2018), however, the small scope of action of biological control does not allow for large-scale replication (Motitsoe et al., 2020; Stenberg et al., 2021). Chemical weed control is inexpensive and widely adapted, but chemical herbicides are poorly utilized during use and pollute soil and water quality (Heap and Duke, 2018; Gould et al., 2018; Peterson et al., 2018; Mennan et al., 2020). Mechanical weeding is less contaminated and more efficient, but intercrop weeds are difficult to remove and have high seedling injury rates, a single weed knife is difficult to meet the weeding requirements of different crops between plants and rows, and mechanical weeding is usually applied at early crop growth stages, which may cause irreversible crop damage (Perez-Ruiz et al., 2012; Zhou et al., 2018; Raja et al., 2020). In recent years, laser weeding has been extended to complex weeding operations. As a precision non-contact physical weeding method, laser weeding has the characteristics of high weeding efficiency, precise positioning, low seedling injury rate and environmental friendliness, so laser weeding was selected for this study.

With the continuous development of weed control research, although several weed control methods have been developed (Teasdale, 1996; Rasmussen, 2004; Harker and O’ Donovan, 2013; Kunz et al., 2018; Alba et al., 2020; Monteiro and Santos, 2022), but still requires a lot of human involvement, low weed control efficiency and cannot do accurate weeding. The application of robots in the field of weed control has improved the efficiency of weeding, it has greatly reduced the labor intensity, reduced the amount of herbicides and to some extent avoided the waste of resources (Perez-Ruiz et al., 2014; Chen et al., 2015; Li et al., 2016; McAllister et al., 2019; Kanagasingham et al., 2020). At present, in agricultural fields, weeding is usually performed by robots carrying chemical herbicides or weed knives. The disadvantages of this method are that there is no recognition of ground weed targets, uniform application when robots carry chemical herbicides, which leads to pesticide contamination, and indiscriminate weeding when they carry weed knives, which leads to crop damage. The introduction of machine vision in the field of weeding has compensated for this shortcoming, and weeding robots with online weed recognition and classification are key contributors to the improvement of correct weeding rates. The use of machine vision to distinguish crop weeds and extract weed coordinates makes weeding targeted and effectively reduces pesticide contamination or crop damage rates.

Chen et al. (2005) studied a direct application weed control robot for controlling weeds in agricultural fields, using end-effectors to cut weeds to apply herbicides, reducing the amount of herbicides used, but the long-term use of chemical herbicides resulted in high weed resistance; Mao et al. (2007) used color features to segment green plants and soil background, position features to detect weeds between rows, and texture features to classify weeds within rows; Zhang et al. (2012) used the advantage of plant G-component values to remove backgrounds such as soil interference, the coordinates of the center of the seedling plant when the seedling hoeing robot advances were determined, but the parameter combination method of image row pixel histogram could not effectively identify small target weeds; Lavania et al. (2015) used the 3D-Otsu method with double threshold processing and cropping rows to classify weeds in corn fields, which is suitable for real-time weed detection; Xiong et al. (2017) developed a static laser weeding robot under fast path planning, which can identify weeds in the laboratory, but the field environment is more complex than the indoor environment, and the reliability of this system for field weeding operations is reduced; Tang et al. (2017) studied k-means preprocessing instead of random initialization of weights in traditional CNN to improve weed identification accuracy; Reiser et al. (2019) developed a motorized in-row rotating weeder where the robot was based on 2D laser scanner data to achieve navigation, which is narrowly applicable to elevated crops such as apple orchards and vineyards; Kanagasingham et al. (2020) effectively enabled the weeding robot to navigate on a prescribed route with integrated GNSS, compass and visual guidance, but accuracy decreases as weed concentration increases; Rakhmatulin and Andreasen (2020) used neural networks to identify weeds and developed an algorithm to estimate laser dose for weed control, but laser energy 1w requires long exposure time and laser energy 5w injures the crop and fails to estimate the optimal laser dose; Xu et al. (2020) developed an algorithm based on absolute characteristic corner point (AFCP) for identifying crop and weed locations to calculate weed density; Ying et al. (2021) used Lightweight Yolov4 was used to accurately detect weeds in carrot fields with an average weed recognition rate of 88.4%. YOLOX is more capable, faster, and more accurate in identifying small targets than previous weed recognition neural networks. YOLOX-L achieved 50.0% AP on the COCO dataset and 68.9 FPS on a single Tesla V100, which It is more in line with real-time image recognition and data processing in the field, so YOLOX was chosen as the god will network for identifying weeds in this experiment.

In this paper, laser weeding is a physical weeding method, which has the advantages of being environmentally friendly, renewable, and harmful to organisms. As most of the previous laser weed control studies have used CO2 laser, which is a red laser with a wavelength of 650-660 mm, the shorter the wavelength means higher energy among different types of lasers. The wavelength of the blue laser selected in this paper is 400-500 mm. The blue laser has been widely used in physics, aviation and other fields because of its short wavelength, small diffraction effect and high energy. Since the blue laser has not been applied to weed control in current research, and the laser weed control effect is related to laser type, laser dose, weed species and growth period, it is necessary to design experiments to find the optimal weed control laser dose according to the above conditions (Mathiassen et al., 2006; Marx et al., 2012). In order to verify the feasibility of blue light laser weed control and the optimal weed control laser dose for this experiment, a blue light laser weed control experiment was designed. In this paper, a YOLOX-based blue light laser weed control robot was designed. Compared with previous laser weed control studies, the mobile platform adopts a tracked chassis to enhance the stability of body movement and rotation, a five-degree-of-freedom actuator was designed to enable precise laser positioning, and the YOLOX neural network was used to identify small targets with significant effect and improve the outdoor weed identification rate.

## 2 Design of robots

### 2.1 General robot description

**Figure 1:**
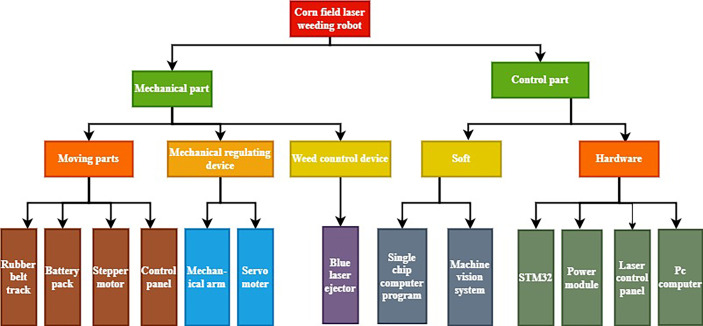
System architecture of laser weeding robot.

The robotic arm laser transmitter, weed crop detection system, communication control system, tracked mobile chassis, and laser transmitter are all components of the laser weeding robot. The robot system architecture is shown in **Figure 1**. A centralized control strategy is adopted, where each subsystem is controlled independently, centralizing management, operation and display, while decentralizing functions, loads and hazards. The central Python (3.7.1) program regulates and collaborates with each subsystem to ensure better real-time control and comprehensive information management in the field.

#### 2.1.1 Design of robot structures

**Figure 2:**
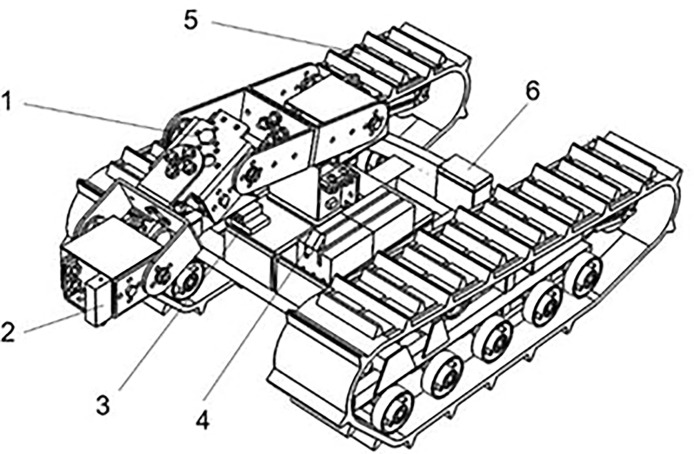
Laser weeding robot. 1. Mechanical arm adjustment mechanism; 2. Blue laser emitter; 3. Line road camera; 4. Identification camera; 5. Crawler mobile chassis; 6. Battery pack.

The overall hardware assembly of the laser weeding robot is shown in **Figure 2**. The image acquisition device includes two cameras, one in the center of the body forward to provide vision navigation for the robot, The camera labeled 3 in **Figure 2**, for example, uses monocular vision and odometer fusion to navigate the robot. The odometer readings are used as auxiliary information to calculate the coordinate position of the feature point in the current robot coordinate system using triangulation. The location of the camera in the real world, is estimated based on the 3D coordinates of the feature point in the current camera coordinate system and its coordinates in the real world, the other in front of the laser emitting robot arm downward to provide real-time images of the weed identification system in the field. The tracked mobile chassis is driven by two stepper motors to drive the symmetrical tracks, which support the whole machine operation through the chassis structure, and the battery pack supplies energy for each part of the robot while balancing the overall weight distribution of the robot. With reference to the planting area of corn field, corn planting row spacing and the mechanical structure of the weeding robot, the robot is designed to have an external dimension of 550×450×400mm (length×width×height), The workable area of the robot arm is 400×400mm, weeding robot weight of 20kg.

#### 2.1.2 Design of a robot’s walking mechanism

The mobile crawler chassis that powers the robot gives it autonomous steering and speed control. In steep plowing and wet, muddy fields, the crawler walking method offers greater stability than the four-wheeled and four-legged walking methods.

**Figure 3:**
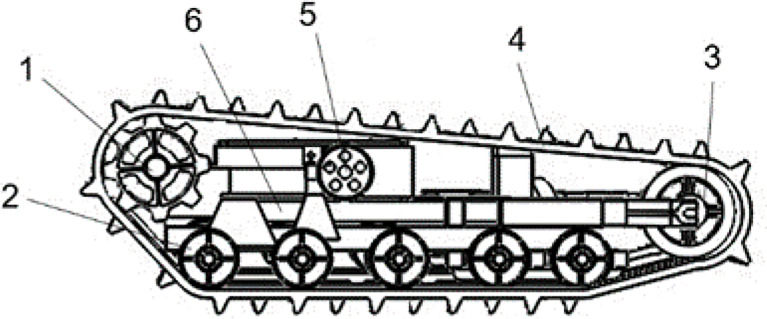
Crawler mobile chassis. 1. Track drive wheels; 2. Support weight running wheels; 3. Guide wheels; 4. Rubber tracks; 5. Carrier wheels; 6. Track tensioning mechanism.

As shown in **Figure 3**, to ensure the stability of the robot in walking and weeding operations, five pairs of support wheels are used at the bottom of the track to support the weight of the robot, increase the force area and reduce damage to the cultivated land. The track must be tensioned to maintain transmission dependability in order to prevent the drive wheels from slipping and derailing when connecting the drive wheels to the track. The carrier wheel’s job is to pull the track’s loose edge downward. The track tensioning mechanism supports the track laterally by adjusting the distance between the supporting wheel and the guide wheel.

**Figure 4:**
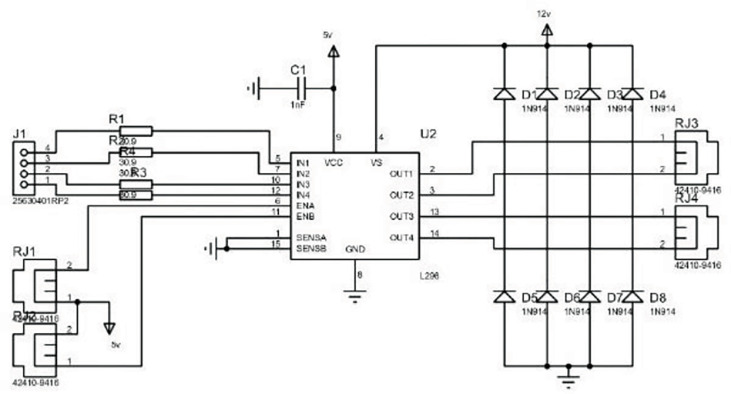
Two motor drives.

The robot is automatically adjusted in the field based on a dual closed-loop system of position and speed parameters to maintain the desired speed and deflection angle. Two servo motors with independent speed and steering are equipped. The track drive wheels transmit the transmission shaft’s torque to the left and right rubber tracks through their protruding teeth, and the rubber tracks’ winding motion propels the weeding robot forward. The servo motors are connected to the track drive wheels through the transmission mechanism to output torque. Emitting pulse is a function of the servo motor itself. To achieve automatic operation and regulation of the circuit, relays are used to automatically regulate the servo motor start, stop, forward, and reverse, as well as to issue control commands and reflect the robot status by turning on and off the circuit. The schematic diagram of the two-way motor drive is shown in **Figure 4**.

### 2.2 Machine Vision

#### 2.2.1 Image acquisition and coordinate transformation

This study relies on a USB camera to collect field picture data in real-time while weeding robots are at work. Ohya et al., 1998 studied A vision-based navigation method for autonomous mobile robots in indoor environments, using monocular cameras for obstacle avoidance navigation. Similar to the previous study, this one employs a single camera to distinguish between crops and weeds (Chen et al., 2021; Sapkota et al., 2022). The weed and corn seedling images were collected at the conservation tillage trial field of Kunming University of Science and Technology. During the weeding operation, the camera pose to the ground was ensured to be the same as the calibrated pose, and the coordinates of the weed’s center position were calculated. The image acquisition device is Sony A6000 digital camera, with effective pixels of about 24.3 million, with Sony E16-50mm OSS lens, the camera aperture set to F8.0. The initial image resolution is 6000×4000 pixels, the initial image size S is 6.0M, the photo aspect ratio is 3:2, the image quality is standard.

**Figure 5:**
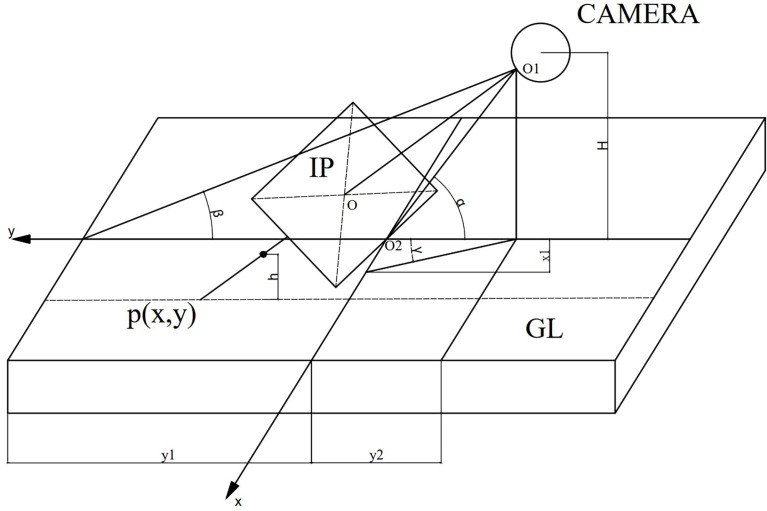
Single visual distance schematic diagram.

During weeding operation, the identification camera fixed in the body obtains real-time image data, and the PC upper computer communicates with the STM32F103 lower computer through the HC-08 Bluetooth serial port to input the image into the weed identification system. And the real coordinates of the corresponding location of the weed plenum are determined using a similar triangle ratio. This monocular camera-ranging model is depicted in **Figure 5** and assumes that the measured weed plenum has an X-axis, Y-axis, and height component. The known quantities are represented in the **Figure 5** by the image coordinate system, the robot camera coordinate system, and the real coordinate system.

The real coordinates of the weed center of mass are calculated in accordance with the triangle similarity theorem, and the image coordinates of the lens centroid, focal length, and pixel aspect are directly solved from the calibration.

Where H is the height of the camera H. *y*_1_ is the distance between the image coordinate center corresponding to the real coordinate point and the camera on the Y-axis. The lens (ucenter, ucenter) is Image coordinates of the center point of. P1(u,0) is Image coordinates of the measured pixel point. xpix is actual pixel length. ypix is actual pixel width. f is camera focal length. The calculation formula is as follows:


$$\begin{array}{c} \alpha =\arctan\left(\frac{H}{y_{2}}\right)\left(1\right) \end{array}$$

$$\begin{array}{c} \gamma =\arctan\left(\frac{x_{1}\times ypix}{f}\right)=\frac{\left(u-ucenter\right)\times ypix}{f}\left(2\right) \end{array}$$

$$\begin{array}{c} \beta =\alpha -\gamma \left(3\right) \end{array}$$

$$\begin{array}{c} O_{2}p=\frac{H}{tan\beta }\left(4\right) \end{array}$$

The coordinate of the vertical direction in the real coordinate system Y= *O*_2_ *P* .

X-axis by Y-axis coordinate scale calculation.


$$\begin{array}{c} O_{1}P_{1}=\sqrt{\left[{\left(\left(u-ucenter\right)\times xpix\right)}^{2}+f^{2}\right]}\left(5\right) \end{array}$$

$$\begin{array}{c} O_{1}P=\frac{H}{sin\beta }\left(6\right) \end{array}$$

$$\begin{array}{c} \frac{PQ}{O_{1}P_{1}}=\frac{O_{1}P}{u}\left(7\right) \end{array}$$

$$\begin{array}{c} PQ=\frac{O_{1}P\times O_{1}P_{1}}{u}\left(8\right) \end{array}$$

Realistic coordinate system vertical direction coordinates X = PQ.

Since the camera is at a certain height from the ground, the coordinates (X,Y) derived above are transformed by projection to obtain the true coordinates $\left(\dot{X},\dot{Y}\right)$ .


$$\begin{array}{c} \left(\dot{X},\dot{Y}\right)=\left(X,Y\right)\times \left(1-\frac{h}{H}\right)\left(9\right) \end{array}$$

The coordinates of the weed centroid can be obtained by combining equation (4), equation (8) and equation (9).

#### 2.2.2 YOLOX-based crop and weed detection

The foundation of the visual weed crop detection system is the generation and collection of datasets (Quan et al., 2019; Zhong et al., 2019; Li et al., 2021), as well as the construction of a YOLOX network structure. The data of this study was collected from the conservation tillage experimental field of Kunming University of Science and Technology. 15,000 photos were collected by Lab team, and photos of weeds and corn seedlings were collected in different weather, time and angles to avoid the effects of light changes, camera angles and crop growth stages on the detection results. Because the pictures were gathered too quickly, there were too many overlapping areas of adjacent pictures and some pictures with poor imaging quality, and these pictures would reduce the recognition effect of the model. Therefore, the dataset was screened and finally 2000 images were selected as the final dataset, and then the dataset was extended to 8400 images by adopting data enhancement such as changing contrast, changing brightness and increasing image noise, etc. The crop and weeds were then calibrated using LabelImg and output in YOLO format. After field survey, weeds in the test field mainly consisted of grass weeds and broadleaf weeds.

The final dataset was divided into two groups: corn and weeds, to reduce the difficulty of robotic arm operation and the amount of time required to generate weed placenames. The specific parameters of the dataset are shown in **Table 1**. 2000 photographs are first chosen as the starting dataset, and 8400 images are added to the enhanced dataset D following data augmentation. In other words, *D*=*S*∪^​^ *T* , *S*∩^​^ *T*=∅ The former is utilized as the training set S and the later as the test T. T is used to assess the model’s test error as an estimate of the generalization error after the model has been trained on S.

**Table 1:** Main parameters of YOLOX network dataset.

Number of species		The number of objects in the dataset	
		maize	weed
The original sample	2000	1049	951
Data enhancement	8400	3745	4646
Training set	6720	2996	3717
Test set	1680	749	909

**Figure 6:**
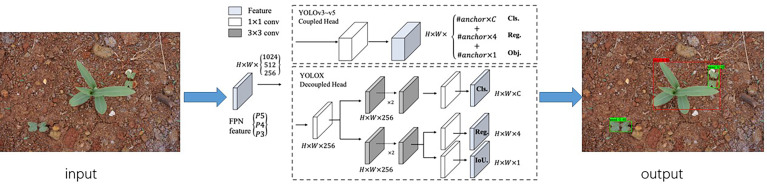
YOLOX maize seedling and weed detection process.

The current target detection networks are Faster ReCNN, YOLO, SSD, DSSD, etc. (Zhao et al., 2018). The YOLO series networks can predict multiple categories and locations at the same time (Tian et al., 2019; Wu et al., 2020). YOLOX has the benefits of real-time performance, accuracy, and minimal processing effort while simultaneously recognizing both large and tiny targets. YOLOX uses YOLOv3-Darknet53 as the network benchmark model, which adds Decoupled Head, SimOTA and other tricks to get YOLOX-Darknet. YOLOX incorporates training methods as EMA weight update, Cosine learning rate mechanism, RandomHorizontalFlip, ColorJitter, and multi-scale data broadening additionally, increasing its detection accuracy over yolov3 by 3%. The network detection process of YOLOX for crops and weeds is shown in **Figure 6**.

#### 2.2.3 Extraction of maize seedlings and weed locations

**Figure 7:**
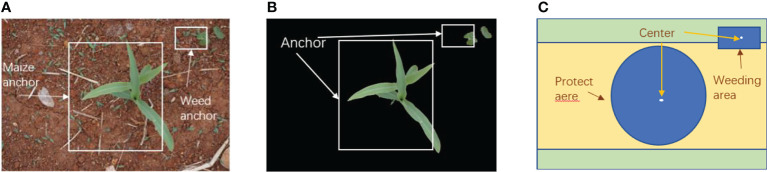
Determination of protected areas and weeding areas **(A)**. Output the anchor box **(B)**. Remove the background **(C)**. Form protected areas and weed areas.

The real-time field image is divided into a weeding operation zone and a seedling protection zone prior to robot operation by the weed identification system. The weeding operation zone and seedling protection zone are originally established by the anchor frame predicted by the target detection. As depicted in **Figure 7**, a protected area and a target weeding area have been established. YOLOX detects corn seedlings and weeds, extracts the location of crop and weed centroids by similar triangle proportional relationship based on the recognition results, and then divides corn seedling protection zone and weeding operation zone (Tang et al., 2017). To calculate the size of the maize protection zone accurately, the maize seedlings’ leaf range must be taken into consideration. In this thesis, maize was weeded at the three-leaf stage, and seedlings from the test field were chosen at random to have their leaf crown radii measured. The radius of the protected area was determined to be 76 mm by taking several measurements of the leaf crown radius, and the radius of the protected area was finally increased to be 80 mm by increasing 4 mm due to human measurement error. The weeding operation area is a circle centered on the turntable of the robot arm with the radius of 400mm for the operation of the robot arm.

#### 2.2.4 Control system and strategy

##### 2.2.4.1 The hardware setup of the control system

The STMF103 microcontroller serves as the primary control chip and the YOLOX detecting network serves as the system’s core for the laser weeding robot. The STMF103 microcontroller provides many I/O ports, strong functionality, and room for future function extension. YOLOX detection is quick and computationally light, which satisfies the technical requirements of real-time field image capture.

##### 2.2.4.2 Methods for developing weed control zones in various circumstances

**Figure 8:**
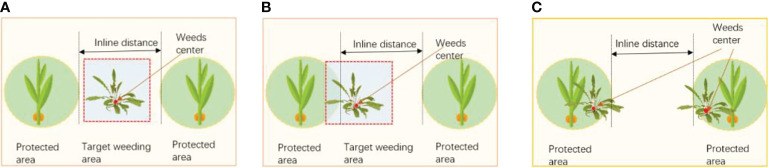
Determine the weeding strategy, **(A)** complete weeding **(B)** partial weeding **(C)** no weeding.

In order to decrease the rate of seedling injury, the system must decide on a weeding strategy and split the weeding operation region according to the weed distribution position in the actual corn field. Weed crossover with maize seedlings was full shade, half shade and no shade, so the weed removal strategy was no removal, partial removal and complete removal, respectively as illustrated in **Figure 8**. The entire eradication approach is used when the weeds are not obstructing the maize seedlings, as shown in **Figure 8A**, and the weeding robot moves on without halting. The partial removal approach is used when the weed center point is outside the corn seedling protection zone, but some of the weed leaves are at the boundary of the corn seedling protection zone, as shown in **Figure 8B**. When the weed is completely covered by the corn seedling, the robot stops to control the laser emitter operation, as shown in **Figure 8C**.

##### 2.2.4.3 Process control for weeding

**Figure 9:**
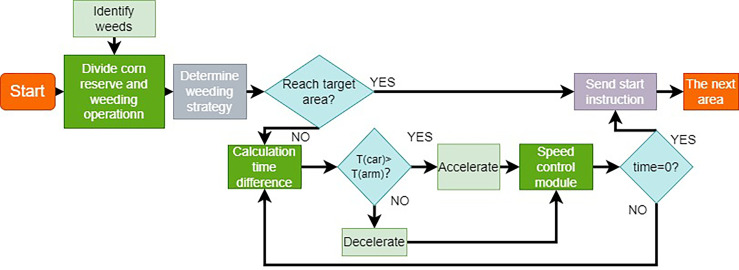
Flow chart of weeding process 1. laser emitter 2. robot arm I 3. robot arm II 4. robot arm III 5. robot arm IV 6. robot arm turntable 7. servo motor.

The mechanical arm alignment to weed centroid duration and real-time journey displacement control are combined in the laser emitter control technique. As indicated in **Figure 9**, The weeding approach is broken down into the following parts: (1) corn seedling protection zone and weeding work area are determined by YOLOX detection network; (2) weeding strategy is determined according to corn seedling and weed distribution; (3) weed center coordinates are sent from weed recognition module to control center; (4) time difference t is determined by trolley travel speed and recognition module calculation speed, if the robot travel time required is faster than the time required for robot arm movement then robot decelerate, if the robot travels too slow then accelerate; (5) when it reaches the target area, send the start command to the control system together with the weed recognition system to control the laser emitter to emit laser; (6) continue to travel into the next target area. The flow chart of weeding process is shown in **Figure 9**.

### 2.3 Robotic arm laser emitter

When designing the robot arm, the laser emitter is adjusted in the horizontal plane after moving above the weed, and its alignment to the center of the weed can meet the operational requirements, so the rotation axis of the six-axis mechanical end is removed. This is since when weeding, the laser emitter just needs to be centered on the weeds, and the five-degree-of-freedom robot arms are simpler to handle, quicker to execute laser emitter modules, and have more precise laser positioning. It also takes less time to calculate the position of the robot arm’s end in the control center.

The spatial mechanism’s degree of freedom F is calculated as:


$$\begin{array}{c} F=6n-5P5-4P4-3P3-2P2-P1\left(10\right) \end{array}$$

where  *n* is the number of active members. *P*5 denotes a member with one degree of freedom and five restrictions, *P*4 , *P*3 , *P*2 , *P*1 , and so on.

**Figure 10:**
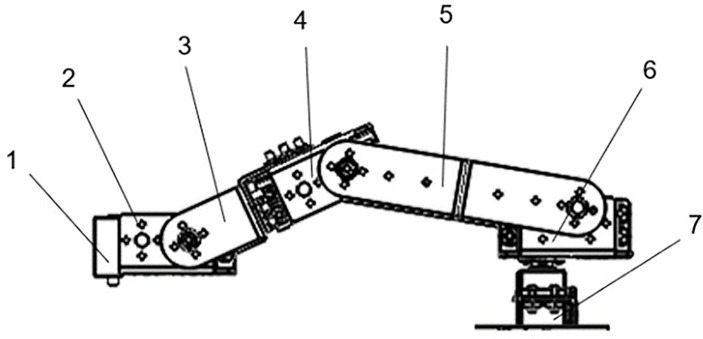
Structural design of five-degree-of-freedom robotic arm. 1. Laser emitter; 2. robot arm I; 3. robot arm II; 4. robot arm III; 5. robot arm IV; 6. robot arm turntable; 7. servo motor.

The robotic arms I to IV and the robotic arm turntable make up the five movable members of the five-degree-of-freedom robotic arm that was designed in this work. Each of the five movable parts has five constraints and one degree of freedom. The specific structure design of the five-degree-of-freedom robot arm is shown in **Figure 10**.

The laser emitter is threaded to the end of the robotic arm I, keeping the operating attitude perpendicular to the ground at all times. The swing is controlled by a servo motor, which is articulated with robotic arm I. Servo motor control articulates and rotates robotic arms II and III. Robotic arm IV is articulated with robotic arm III and robotic arm turntable, and it is controlled by a servo motor to swing with robotic arm III. Two servo motors control the swing of robot arm IV and the robot arm turntable, and the robot arm turntable is hinged on the entire machine frame, with the servo motor controlling its rotation relative to the frame. The robot arm turntable is in charge of driving the entire robot arm. The servo motor model HM-MS10 is selected according to the mechanical load and characteristics of the robot arm. Its performance parameters are operating voltage 5V, pulse width range 500~2500us, mechanism limit angle 180°, response speed 0.7sec·60degree^-1^, pull force 9.4kg·cm^-1^. The PWM pulse width of 2.5ms corresponds to the servo motor’s angle range of 0° to 180°, and the servo motor adjusts the angle with a duty cycle of 0.5ms, the control accuracy can reach 0.3° in the range of 2000 pulse widths. This motor can meet the operational requirements of robotic arm weeding.

**Figure 11:**
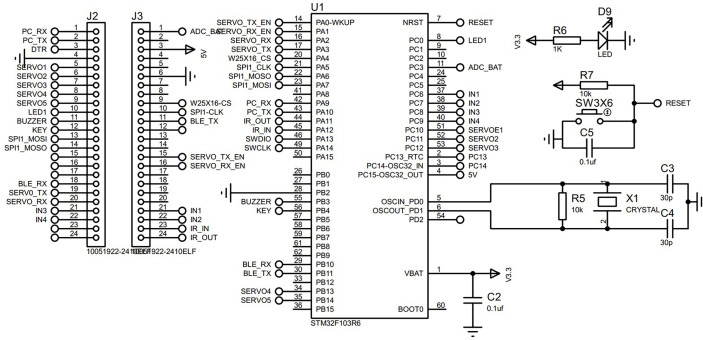
Schematic of the controller working principle.

A movable platform-mounted robotic arm with five degrees of freedom, a laser emitter, and an associated control system make up the robotic arm laser emitter module. The robotic arm is propelled by five servo motors and has five degrees of freedom. The host computer issues control commands to the controller, which moves the robotic arm as directed. The STM32F103 microcontroller that powers the robot arm can regulate the movement of the crawler moving platform and transmit the angle values for each axis to the robot arm’s top computer. HC-08 The STM32 microcontroller of the robot arm controller and the higher computer of the remote control communicate with each other *via* Bluetooth serial connection. The working schematic diagram of the controller is shown in **Figure 11**.

### 2.4 Validation of blue light laser weed control

Based on the study by Streibig et al. in 1993 on laser dose and plant dry weight after laser action, the change in dry weight of weeds after laser action can be described by equation (11).


$$\begin{array}{c} DW=C+\frac{D-C}{1+{\left(\frac{\mathrm{lg}\left(DOSE+1\right)}{\mathrm{lg}\left(ED_{50}+1\right)}\right)}^{B}}+\delta \left(11\right) \end{array}$$

Where DW is the dry weight of weed after laser action. Dose is the laser dose. C is the minimum dry weight mass of the weed after laser action. D is the maximum dry weight mass of weed without laser action. $1+{\left(\frac{\mathrm{lg}\left(DOSE+1\right)}{\mathrm{lg}\left(ED_{50}+1\right)}\right)}^{B}$ is used to describe the position and inclination angle of the laser dose and dry weight curve when the dry weight of weed is reduced to 50% of D. σ is an error of approximately zero.

Streibig et al. showed that as the laser dose increased, the dry weight DW of the plant body was an S-shaped decreasing curve with a value of approximately 0 for the minimum dry weight mass C. When the laser dose tended to infinity, the value of weed dry weight DW was approximately equal to the minimum dry weight mass C, indicating that the high dose of laser irradiation killed the plant and caused the dry weight of the plant to tend to 0. When no laser irradiation was used and the value of laser dose was 0, the value of weed dry weight DW was approximately equal to the maximum dry weight mass D, indicating that the plant grew normally without laser irradiation. Laser irradiation and the value of laser dose was 0, the value of weed dry weight DW was approximately equal to the maximum dry weight mass D, indicating that the plants grew normally without laser irradiation.

The efficiency of the blue laser on the dry weight of weeds during this growth period was determined after a given amount of time by calculating the difference between the dry weight of weeds in the test area and the dry weight of weeds in the blank region without laser irradiation (Dudareva et al., 2020; Rani et al., 2022).


$$\begin{array}{c} Y=\frac{CK-E}{CK}\times 100\%\left(12\right) \end{array}$$

where *Y* is the dry weight control effect. *CK* is the dry weight of weeds in the blank control area without laser irradiation. *E* is the dry weight of weeds in the laser-irradiated test area.

**Figure 12:**
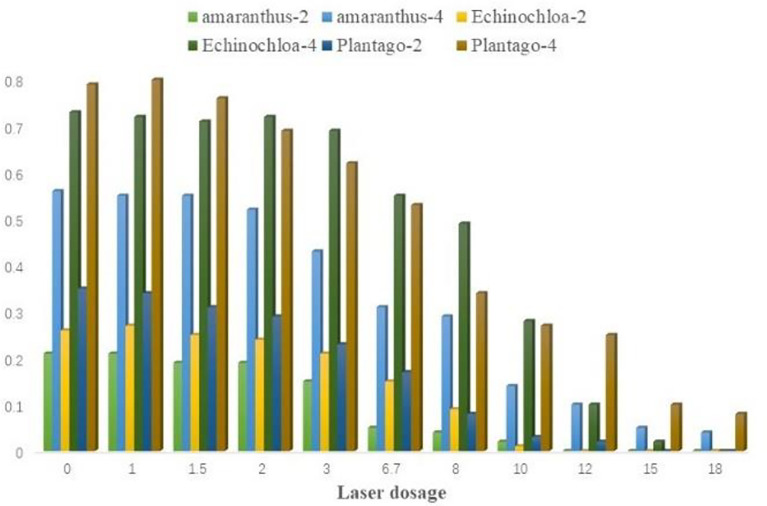
Effect drawing of laser irradiation.

To determine the most effective weed control laser dose, three common maize-associated weeds (Echinochloa colonum, Amaranthus retroflexus, Plantago asiatica) were chosen for laser weed control studies. The weeds were preserved in their original growing habitat following the laser irradiation test and continued to be grown in the conservation tillage test field at the Kunming University of Science and Technology. The weeds were chopped off above the surface with scissors after ten days, and they were then dried in an oven at a continuous temperature of 85 degrees C for ten hours. Using an electronic balance, the dry weight of the weeds above the surface was calculated and recorded. The laser irradiation test is shown in **Figure 12**.

**Figure 13:**
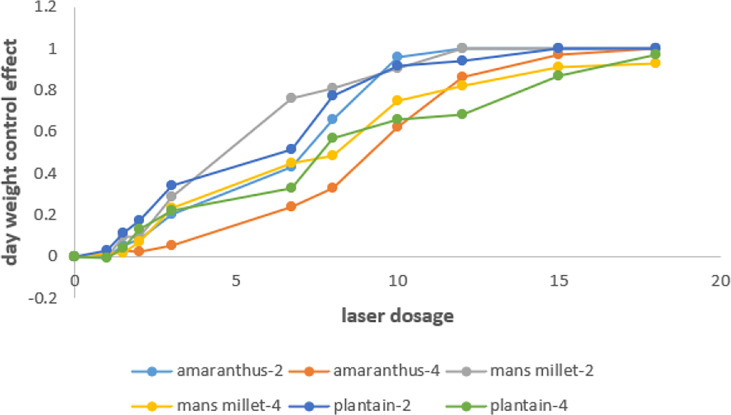
Effect diagram of laser irradiation dry weight.

Separate calculations of the three weeds’ dry weight control effectiveness following laser studies at the two-leaf and four-leaf phases are shown in **Figure 13**.

The expected dry weight control value was 85%. The average values of the dry weight control efficiency of the three experimental weeds at the two and four leaf stages were taken, and then the curve was fitted. The fitted curve of Amaranthus retroflexus is obtained as:


$$y_{1}=-0.0002x^{3}+0.0008x^{2}+0.0946x-0.0574\;R^{2}=0.9925$$

$$x_{1}=13.9J\cdot mm^{-1}$$

Fitted curves of Echinochloa colonum:


$$y_{2=}-0.0006x^{3}+0.0134x^{2}-0.0072x+0.0097\;R^{2}=0.9913$$

$$x_{2}=14.5J\cdot mm^{-1}$$

Fitted curves of Plantago asiatica:


$$y_{3}=-0.00006x^{3}-0.0049x^{2}+0.1268x-0.0652\;R^{2}=0.9919$$

$$x_{3}=12.1J\cdot mm^{-1}$$

The test results showed that the blue laser could effectively inhibit the regrowth of the three weeds in this test, and the dry weight control efficiency of the blue laser at high doses exceeded 90% for all three weeds. The blue light laser could kill the seedlings of Echinochloa colonum in two growth periods and effectively control the seedlings of Amaranthus retroflexus and Plantago asiatica, which are narrow-leaved weeds of the grass family, and both Amaranthus retroflexus and Plantago asiatica are broad-leaved weeds. Based on the curve fitting results of the weeding test, the laser emission doses of the robotic weeding control system in this paper were set to 13 J/mm and 15 J/mm for grassy weeds and broadleaf weeds, respectively.

## 3 Field trials

The field trial was conducted in the conservation tillage experimental field of Kunming University of Science and Technology. The test subjects were maize seedlings and the weeds that were present around 20 days after sowing. The test field’s maize plants were spaced 30 cm apart, and its rows were 70 cm apart.

### 3.1 Robot vision system test

The recognition rate of weeds and corn seedlings was assessed for weeding robot vision system individually. The host computer compressed the data uploaded from the USB camera into a video with a picture quality of 640 × 640 and a frame rate of 10 frames per second, and the weeding robot was manually controlled to move at a constant speed through the field. Real-time item detection from the input video was provided by the robot vision system, together with categorization and coordinates for each thing found.

**Figure 14:**
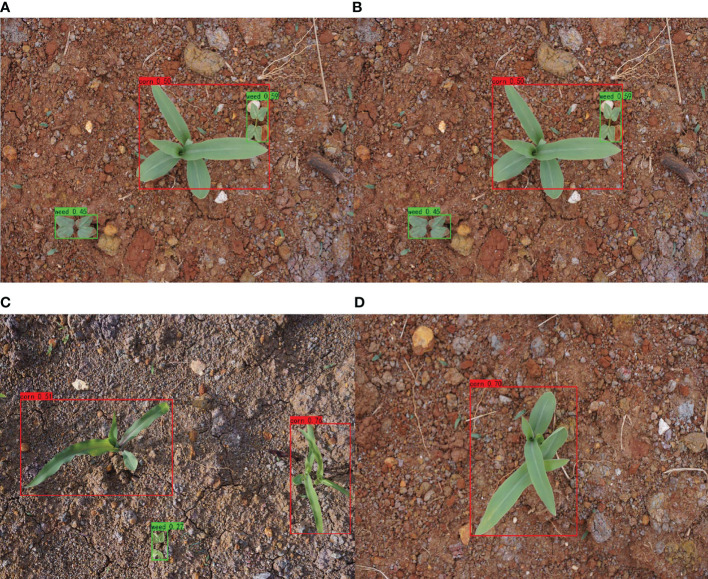
Visual system test result diagram.

The target detection method is challenging because weeds and corn seedlings are distributed differently. The tested model was put to the test with various corn weed distributions. The visual system test result diagram is shown in **Figure 14**.

The prototype was utilized in the test field to evaluate the effectiveness of the robotic vision system in detecting weeds in real-time at two travel speeds. The configuration of the top computer in the experiment was identical to the hardware used in training the algorithm model. The test results are shown in **Tables 2**, **3** below.

**Table 2:** Detection results of maize seedlings by weeding robot prototype.

Robot speed	Corn row serial number	Actual number of corn seedlings	Number of successful identifications	Average detection rate
0.2m/s	1	67	61	92.45%
	2	63	58	
	3	68	62	
	4	67	64	
0.3m/s	1	63	56	89.15%
	2	68	60	
	3	63	57	
	4	64	57	
0.4m/s	1	61	53	88.35%
	2	66	58	
	3	63	56	
	4	59	53	

**Table 3:** Detection results of weeds by weeding robot prototype.

Robot speed	Weed row serial number	Actual number of weeds	Number of successful identifications	Average detection rate
0.2 m/s	1	52	45	88.94%
	2	54	50	
	3	50	44	
	4	52	46	
0.3m/s	1	52	43	84.6%
	2	54	46	
	3	50	44	
	4	52	43	
0.4m/s	1	52	43	84.13%
	2	54	45	
	3	50	43	
	4	52	44	

### 3.2 Robotic weeding trials

**Figure 15:**
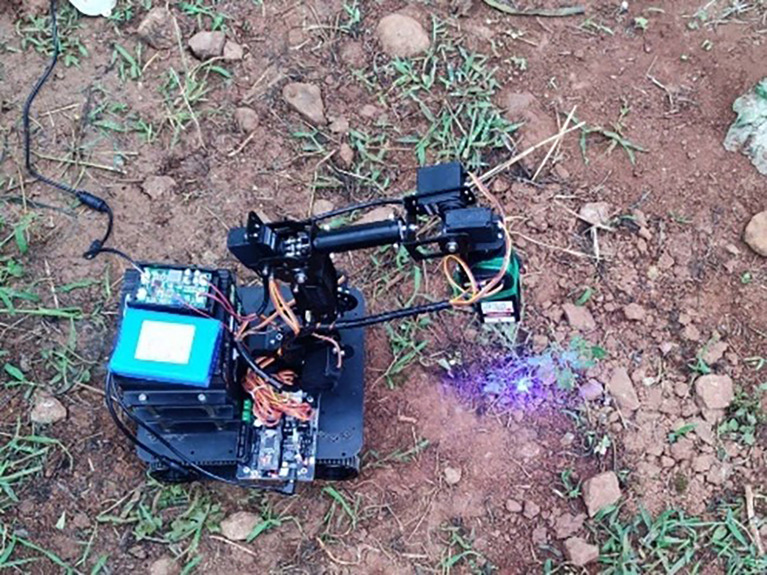
Robot weeding operation site.

The “stop&go” weeding strategy was adopted in this field trial, which can avoid the problem of the mechanical arm adjusting mechanism and the walking mechanism having relative displacement and failing to align with the weed centercenter, and improve the weeding rate. In the field test, when the robot vision system successfully detected the weeds and met the weeding conditions, the robot slowed down and stopped at the weed position. Then sent the weeding command to the control center in the stationary state, and the robot arm moved to the top of the weeds to complete the weeding operation. Robot weeding operation site as shown in **Figure 15**.

The average weed dry weight control efficiency and the average seedling injury rate served as the test indices. Surface levelness was the test factor, and the test was conducted by first measuring the surface levelness. When the test is first measured the surface flatness, take the horizontal reference line on the highest point of the surface, divide it into 10 equal parts, and measure the distance from each equal part to the highest horizontal line of the surface. Calculate the average value and standard deviation, and express the surface flatness of the distance by standard deviation. In the test, two parts of the maize planting field were chosen, each of which comprised three rows of maize planting with a row length of roughly 6 m. The first aliquot was taken at intervals of 60 cm, and the surface flatness was estimated by measuring the distance between the aliquot and the surface (GB/T5668-2008).


$$\begin{array}{c} P=\sqrt{\frac{\sum_{i=0}^{n}{\left(d_{i}-d\right)}^{2}}{n-1}}\left(13\right) \end{array}$$

Where *P*  is ground levelness, *d*_*i*_ is vertical distance of the i-th measurement point in the field from the fitted plane, *d* is Average of vertical distance of all measurement points from the surface.

The test results showed that the surface of the first, second, and third operation rows was relatively flat, measuring 3.9 cm, 4.7 cm, and 3.7 cm, respectively, and being less than 5 cm; the surface gullies of the fourth, fifth, and sixth operation rows were greater, measuring 8.3 cm, 9.6 cm, and 8.9 cm, respectively, and being between 5 cm and 10 cm. The results of the robot’s field weeding trials are shown in **Table 4**.

**Table 4:** Field weeding effect of weeding robot prototype.

Surface flatness	Row number	Number of weeds	Number of corn seedlings	Number of injured seedlings	Average weed dry weight control efficiency	Average injury rate of seedlings
≤5cm	1	24	23	1	85%	4.68%
	2	27	21	1		
	3	27	20	1		
≤7.5cm	4	26	19	1	81.96%	4.92%
	5	27	22	2		
	5	24	20	0		
≤10cm	4	25	20	1	80.07%	5.08%
	5	26	18	0		
	6	25	21	2		

## 4 Results and discussion

In the experiment of verifying the feasibility of blue light laser weed control and determining the laser dose, the laser emission doses of grass weeds and broadleaf weeds in this experiment were set as 13J/mm and 15J/mm respectively under the premise that the dry weight control effect of weeds reached 85%. The laser dose required to remove grass weeds was lower than that of broadleaf weeds. This is due to the shallower roots and narrower leaves of grasses in comparison, which inhibit growth to a higher degree after laser irradiation.

**Table 2** shows that the recognition rate of maize seedlings reached 92.45%, 89.1% and 88.35% at 0.2m/s, 0.3m/s and 0.4m/s of the weeding robot speed, respectively, while **Table 3** shows that the weed recognition rate was 88.94%, 84.6% and 84.13% at 0.2m/s, 0.3m/s and 0.4m/s of the weeding robot speed, respectively. The high crop recognition rate is higher than the weed recognition rate probably because there are more types of weeds in the field, and the data set was constructed with only common weeds and corn seedlings in the corn field, and the algorithm was not sufficiently trained for some types of weeds, while the shape characteristics of corn seedlings are similar, and there are many types of weeds with different shapes, and the detection difficulty of weeds is higher than that of corn; with the increase of robot driving speed, both corn seedling recognition rate and weed recognition rate decreased, which may be due to the fact that the image quality captured by the recognition camera at high speed of the robot is lower than that of the image quality at low speed.

**Table 4** shows that the dry weight control efficiency of weed control was 85%, 81.9%, 80.07%, and seedling injury rate was 4.68%, 4.92%, and 5.08% in the field test under the conditions of ≤5cm, ≤7.5cm, and ≤10cm respectively for the slope of monopoly furrow, and 9 groups of different slope tests showed that the dry weight control efficiency of weeds decreased and seedling injury rate increased with the increase of slope of cultivated land. This may be due to the fact that the laser emitter could not accurately aim at the weed center when the robot passed through the uneven ground; the laser beam emitted to the weed surface spot area became large energy dispersion; the height of the recognition camera and the ground changed when the body was tilted, and the image weed center coordinates were mapped to the real world with some deviation in the process.

Quan et al. (2021) used deep learning combined with directional weeding tools in 2018, and the average rate of sloppy removal was 85.91%. Cubero et al. (2020) used machine vision to control the electrode discharge of the end-effector of a 6-DOF robotic arm to kill weeds, and the accuracy rate of weed localization reached 84%. The experimental results show that the weeding effect of the weeding robot designed in this paper is better than the above research, and the weeding robot has achieved better maize seedling recognition rate and weed recognition rate. The interference factors in the field experiment were higher than those in the laboratory experiment, but the weed recognition rate and crop recognition rate were still 88.94% and 92.45%, respectively, which were higher than those in the above experiment.

## 5 Conclusion

This study demonstrates the field weeding capability and detection of corn seedlings and weeds (grass and broadleaf weeds) of the blue laser weeding robot, with better detection results of the YOLOX network for weed identification compared to previous convolutional neural networks; reduced control difficulty and robot positioning time of the five-degree-of-freedom robot arm compared to the six-degree-of-freedom robot arm; and compared to the traditional weeding robot. Using machine vision to locate weeds has stronger robustness, reliability and lower injury second rate than traditional weeding robots. The results show that the weeding robot can effectively remove different kinds of weeds from corn seedling fields.

## Data availability statement

The raw data supporting the conclusions of this article will be made available by the authors, without undue reservation.

## Author contributions

HBZ and LB conceived and planned the experiments. YZ and DM carried out the experiments and contributed to sample preparation. HL, HZ, HBZ and LB contributed to the interpretation of the results. YZ processed the experimental data and wrote the manuscript with input from all authors. DM, HL, HZ, LB, HBZ contributed to the final version of the manuscript. HBZ supervised the project. All authors provided critical feedback and helped shape the research. All authors contributed to the article and approved the submitted version.

## Funding

This work was supported by the National Natural Science Foundation of China (Grant No. 51865022).

## Acknowledgments

We would like to thank the National Fund Committee for its financial support, Xian Wu for acquisition of the maize images, Tao Huang and Guanyu Guo for preparation of the weeds.

## Conflict of interest

The authors declare that the research was conducted in the absence of any commercial or financial relationships that could be construed as a potential conflict of interest.

## Publisher’s note

All claims expressed in this article are solely those of the authors and do not necessarily represent those of their affiliated organizations, or those of the publisher, the editors and the reviewers. Any product that may be evaluated in this article, or claim that may be made by its manufacturer, is not guaranteed or endorsed by the publisher.

## References

[B1] Ab Rahman S. F. S. Singh E. Pieterse C. M. J. Schenk P. M. (2018). Emerging microbial biocontrol strategies for plant pathogens. Plant Sci. 267, 102–111. doi: 10.1016/j.plantsci.2017.11.012 29362088

[B2] Alba O. S. Syrovy L. D. Duddu H. S. N. Shirtliffe S. J. (2020). Increased seeding rate and multiple methods of mechanical weed control reduce weed biomass in a poorly competitive organic crop. Field Crops Res. 245. doi: 10.1016/j.fcr.2019.107648

[B3] Barratt B. I. P. Moran V. C. Bigler F. van Lenteren J. C. (2018). The status of biological control and recommendations for improving uptake for the future. Biocontrol 63, 155–167. doi: 10.1007/s10526-017-9831-y

[B4] Chen Y. Tian L. Zheng J. (2005). Development of weeding robot based on direct herbicide application method. Trans. Chin. Soc. Agric. Machinery 36 (10), 91–93,129.

[B5] Chen Y. Wu Z. Zhao B. Fan C. Shi S. (2021). Weed and corn seedling detection in field based on multi feature fusion and support vector machine. Sensors 21 (1). doi: 10.3390/s21010212 PMC7796182 33396255

[B6] Chen Z. Zhang C. Li N. Sun Z. Li W. Zhang B. (2015). Study review and analysis of high performance intra-row weeding robot. Trans. Chin. Soc. Agric. Eng. 31 (5), 1–8.

[B7] Cubero S. Marco-Noales E. Aleixos N. Barbe S. Blasco J. (2020). RobHortic: A field robot to detect pests and diseases in horticultural crops by proximal sensing. Agriculture-Basel 10 (7). doi: 10.3390/agriculture10070276

[B8] Dudareva L. Tarasenko V. Rudikovskaya E. (2020). Involvement of photoprotective compounds of a phenolic nature in the response ofArabidopsis ThalianaLeaf tissues to low-intensity laser radiation. Photochem. Photobiol. 96 (6), 1243–1250. doi: 10.1111/php.13289 32474931

[B9] Gould F. Brown Z. S. Kuzma J. (2018). Wicked evolution: Can we address the sociobiological dilemma of pesticide resistance? Science 360, 728–732. doi: 10.1126/science.aar3780 29773742

[B10] Harker K. N. O'Donovan J. T. (2013). Recent weed control, weed management, and integrated weed management. Weed Technol. 27 (1), 1–11. doi: 10.1614/WT-D-12-00109.1

[B11] Heap I. Duke S. O. (2018). Overview of glyphosate-resistant weeds worldwide. Pest Manage. Sci. 74, 1040–10049. doi: 10.1002/ps.4760 29024306

[B12] Kanagasingham S. Ekpanyapong M. Chaihan R. (2020). Integrating machine vision-based row guidance with GPS and compass-based routing to achieve autonomous navigation for a rice field weeding robot. Precis. Agric. 21 (4), 831–855. doi: 10.1007/s11119-019-09697-z

[B13] Kunz C. Weber J. F. Peteinatos G. G. Sokefeld M. Gerhards R. (2018). Camera steered mechanical weed control in sugar beet, maize and soybean. Precis. Agric. 19 (4), 708–720. doi: 10.1007/s11119-017-9551-4

[B14] Lavania S. Matey P. S. Ieee (2015). Novel method for weed classification in maize field using Otsu and PCA implementation. In Paper presented at the 2015 IEEE International Conference on Computational Intelligence and Communication Technology CICT 2015. (534–537). Proceedings Paper retrieved from: doi: 10.1109/CICT.2015.71

[B15] Li Y. Al-Sarayreh M. Irie K. Hackell D. Bourdot G. Reis M. M.. (2021). Identification of weeds based on hyperspectral imaging and machine learning. Front. Plant Sci. 11. doi: 10.3389/fpls.2020.611622 PMC7868399 33569069

[B16] Li N. Chen Z. Zhu C. Zhang C. Sun Z. Li W. (2016). System design and experiment of electric driven weeding robot. Trans. Chin. Soc. Agric. Machinery 47 (5), 15–20,69.

[B17] Mao W. Cao J. Jiang H. Wang Y. Zhang X. (2007). In-field weed detection method based on multi-features. Trans. Chin. Soc. Agric. Eng. 23 (11), 206–209.

[B18] Marx C. Barcikowski S. Hustedt M. Haferkamp H. Rath T. (2012). Design and application of a weed damage model for laser-based weed control. Biosyst. Eng. 113 (2), 148–157. doi: 10.1016/j.biosystemseng.2012.07.002

[B19] Mathiassen S. K. Bak T. Christensen S. Kudsk P. (2006). The effect of laser treatment as a weed control method. Biosyst. Eng. 95 (4), 497–505. doi: 10.1016/j.biosystemseng.2006.08.010

[B20] McAllister W. Osipychev D. Davis A. Chowdhary G. (2019). Agbots: Weeding a field with a team of autonomous robots. Comput. Electron. Agric. 163. doi: 10.1016/j.compag.2019.05.036

[B21] Mennan H. Jabran K. Zandstra B. H. Pala F. (2020). Non-chemical weed management in vegetables by using cover crops: A review. Agronomy-Basel 10 (2). doi: 10.3390/agronomy10020257

[B22] Monteiro A. Santos S. (2022). Sustainable approach to weed management: The role of precision weed management. Agronomy-Basel 12 (1). doi: 10.3390/agronomy12010118

[B23] Motitsoe S. N. Coetzee J. A. Hill J. M. Hill M. P. (2020). Biological control of salvinia molesta (DS Mitchell) drives aquatic ecosystem recovery. diversity-basel 12 (5). doi: 10.3390/d12050204

[B24] Ohya A. Kosaka A. Kak A. (1998). Vision-based navigation by a mobile robot with obstacle avoidance using single-camera vision and ultrasonic sensing. IEEE Trans. Robotics Automation 14 (6), 969–978. doi: 10.1109/70.736780

[B25] Perez-Ruiz M. Slaughter D. C. Fathallah F. A. Gliever C. J. Miller B. J. (2014). Co-Robotic intra-row weed control system. Biosyst. Eng. 126, 45–55. doi: 10.1016/j.biosystemseng.2014.07.009

[B26] Perez-Ruiz M. Slaughter D. C. Gliever C. J. Upadhyaya S. K. (2012). Automatic GPS-based intra-row weed knife control system for transplanted row crops. Comput. Electron. Agricult. 80, 41–49. doi: 10.1016/j.compag.2011.10.006

[B27] Peterson M. A. Collavo A. Ovejero R. Shivrain V. Walsh M. J. (2018). The challenge of herbicide resistance around the world: a current summary. Pest Manage. Science 74, 2246–2259. doi: 10.1002/ps.4821 29222931

[B28] Quan L. Feng H. Li Y. Wang Q. Zhang C. Liu J.. (2019). Maize seedling detection under different growth stages and complex field environments based on an improved faster r-CNN. Biosyst. Eng. 184, 1–23. doi: 10.1016/j.biosystemseng.2019.05.002

[B29] Quan L. Zhang J. Jiang W. Li H. Yang C. Zhang X. (2021). Development and experiment of intra-row weeding robot system based on protection of maize root system. Trans. Chin. Soc. Agric. Machinery 52 (12), 115–123.

[B30] Raja R. Nguyen T. T. Slaughter D. C. Fennimore S. A. (2020). Real-time weed-crop classification and localisation technique for robotic weed control in lettuce. Biosyst. Eng. 192, 257–274. doi: 10.1016/j.biosystemseng.2020.02.002

[B31] Rajcan I. Swanton C. J. (2001). Understanding maize-weed competition: resource competition, light quality and the whole plant. Field Crops Res. 71, 139–150. doi: 10.1016/S0378-4290(01)00159-9

[B32] Rakhmatulin I. Andreasen C. (2020). A concept of a compact and inexpensive device for controlling weeds with laser beams. Agronomy-Basel 10 (10). doi: 10.3390/agronomy10101616

[B33] Rani B. S. Chandrika V. Reddy G. P. Sudhakar P. Nagamadhuri K. V. Sagar G. K. (2022). Residual effect of weed management practices executed in preceding maize on succeeding greengram. Legume Res. 45 (5), 631–638. doi: 10.18805/LR-4477

[B34] Rasmussen I. A. (2004). The effect of sowing date, stale seedbed, row width and mechanical weed control on weeds and yields of organic winter wheat. Weed Res. 44 (1), 12–20. doi: 10.1046/j.1365-3180.2003.00367.x

[B35] Reiser D. Sehsah E.-S. Bumann O. Morhard J. Griepentrog H. W. (2019). Development of an autonomous electric robot implement for intra-row weeding in vineyards. Agriculture-Basel 9 (1). doi: 10.3390/agriculture9010018

[B36] Sapkota B. B. Hu C. Bagavathiannan M. V. (2022). Evaluating cross-applicability of weed detection models across different crops in similar production environments. Front. Plant Sci. 13. doi: 10.3389/fpls.2022.837726 PMC9096552 35574075

[B37] Stenberg J. A. Sundh I. Becher P. G. Bjorkman C. Dubey M. Egan P. A.. (2021). When is it biological control? a framework of definitions, mechanisms, and classifications. J. Pest Sci. 94 (3), 677–677. doi: 10.1007/s10340-021-01386-z

[B38] Tang J. Wang D. Zhang Z. He L. Xin J. Xu Y. (2017). Weed identification based on K-means feature learning combined with convolutional neural network. Comput. Electron. Agric. 135, 63–70. doi: 10.1016/j.compag.2017.01.001

[B39] Teasdale J. R. (1996). Contribution of cover crops to weed management in sustainable agricultural systems. J. Production Agric. 9 (4), 475–479. doi: 10.2134/jpa1996.0475

[B40] Tian Y. Yang G. Wang Z. Wang H. Li E. Liang Z. (2019). Apple detection during different growth stages in orchards using the improved YOLO-V3 model. Comput. Electron. Agric. 157, 417–426. doi: 10.1016/j.compag.2019.01.012

[B41] Westwood J. H. Charudattan R. Duke S. O. Fennimore S. A. Marrone P. Slaughter D. C.. (2018). Weed Management in 2050: Perspectives on the Future of Weed Science. Weed Sci. 66 (3), 275–285. doi: 10.1017/wsc.2017.78

[B42] Wu D. Lv S. Jiang M. Song H. (2020). Using channel pruning-based YOLO v4 deep learning algorithm for the real-time and accurate detection of apple flowers in natural environments. Comput. Electron. Agric. 178. doi: 10.1016/j.compag.2020.105742

[B43] Xiong Y. Ge Y. Liang Y. Blackmore S. (2017). Development of a prototype robot and fast path-planning algorithm for static laser weeding. Comput. EL Agric. 142, 494–503. doi: 10.1016/j.compag.2017.11.023

[B44] Xu Y. L. He R. Gao Z. M. Li C. X. Zhai Y. T. Jiao Y. B. (2020). Weed density detection method based on absolute feature corner points in field. Agronomy-Basel 10 (1). doi: 10.3390/agronomy10010113

[B45] Ying B. Y. Xu Y. C. Zhang S. Shi Y. G. Liu L. (2021). Weed detection in images of carrot fields based on improved YOLO v4. Traitement Du Signal 38 (2), 341–348. doi: 10.18280/ts.380211

[B46] Zhang C. Huang X. Liu W. Zhang Y. Li N. Zhang J.. (2012). Information acquisition method for mechanical intra-row weeding robot. Trans. Chin. Soc. Agric. Eng. 28 (9), 142–146.

[B47] Zhao H. Qi X. Shen X. Shi J. Jia J. (2018). ICNet for real-time semantic segmentation on high-resolution images in Paper presented at the COMPUTER VISION - ECCV 2018, (418–434), PT III. doi: 10.1007/978-3-030-01219-9_25

[B48] Zhong L. Hu L. Zhou H. (2019). Deep learning based multi-temporal crop classification. Remote Sens. Environ. 221, 430–443. doi: 10.1016/j.rse.2018.11.032

[B49] Zhou F. Wang W. Li X. Tang Z. (2018). Design and experiment of cam rocker swing intra-row weeding device for maize. Trans. Chin. Soc. Agric. Machinery 49 (1), 77–85.

